# Growth Performance, Nutrient Digestibility, Hematological Parameters, and Hepatic Oxidative Stress Response in Juvenile Nile Tilapia, *Oreochromis niloticus*, Fed Carbohydrates of Different Complexities

**DOI:** 10.3390/ani10101913

**Published:** 2020-10-19

**Authors:** Mohamed S. Azaza, Saber A. Saidi, Mohamed N. Dhraief, Abdelfattah EL-feki

**Affiliations:** 1 Aquaculture Laboratory (LR16INSTM03), National Institute of Marine Sciences and Technologies, Salammbo 2025, Tunisia; naceur.dhraief@instm.rnrt.tn; 2 Department of Biology, Faculty of Sciences and Arts-Khulis, University of Jeddah, Jeddah 23218, Saudi Arabia; saberabdelkader@gmail.com; 3 Laboratory of Animal Ecophysiology, Department of Life Sciences Sciences Faculty of Sfax, P.O. Box 1171, Sfax 3000, Tunisia; abdelfattah.elfeki@fss.rnu.tn

**Keywords:** feed nutrition, carbohydrate complexity, growth performance, digestibility, hematological parameters, hepatic antioxidant enzymes, health status, *Oreochromis niloticus*

## Abstract

**Simple Summary:**

Proteins are the highest-cost nutrients of aquafeed and are the essential components in diets and, thus, are acknowledged as the most critical input in aquafeed. The utilization of alternative sources to prepare cheaper feed is requisite to ensure the sustainability of the aquaculture sector. Carbohydrates are the least-expensive form of energy source in practical diet ingredients, more available than proteins, and efficiently used by omnivorous warm-water fishes. The use of carbohydrate-based diets has the advantage of being economically efficient, owing that fish would efficiency utilize the low-cost carbohydrate. Factors that affect the carbohydrate utilization efficiency are carbohydrate origin, dietary level, physical state, technological treatments, and molecular complexity. Also, they can adversely affect fish health through metabolic disorder and physio-clinical signs such as hyperglycemia, increment of glycogen deposition, liver hypertrophy, and histopathological development. Physiological and biochemical characteristics are recognized as a useful tool in the evaluation of the metabolic functions, health status and welfare of farmed species. With reference to our study, knowledge of the physiological and health implication of dietary carbohydrate helps fish nutritionists to tailor and improve the nutritional profile of the diet and hence to provide more adequate and healthy diets for fish.

**Abstract:**

A 45-day feeding trial was conducted to assess the capacity of juvenile Nile tilapia (2.12 ± 0.02 g) to utilize different sources of carbohydrate in their diets. Growth performance, nutrient digestibility, hematological parameters, and hepatic oxidative stress were evaluated. Four experimental diets were formulated to be isonitrogenous (25% crude protein) and isolipidic (10% crude lipid), each containing 20% glucose (GLU-diet), maltose (MAL-diet), dextrin (DEX-diet), and corn starch (CST-diet), respectively. At the end of feeding trial, survival in all groups was above 90% and was not significantly different among groups. The results indicated that fish fed the DEX-diet and CSTA-diet showed significantly (*p* < 0.05) better specific growth rate (SGR), feed conversion ratio (FCR), and protein efficiency ratio (PER) compared with those fed the other diets. The dry matter and carbohydrate digestibility were significantly higher (*p* < 0.05) in groups fed on dextrin and corn starch diets. However, the digestibility of crude protein and energy in diets did not differ significantly (*p* > 0.05) among groups fed on experimental diets. The activities of analyzed antioxidant enzymes in the liver were significantly (*p* < 0.01) higher in groups fed on glucose and maltose diets when compared to other groups. Hematological parameters were affected by the dietary carbohydrate sources; there was a significant increase in hematocrit (Ht), hemoglobin (Hb), and mean corpuscular volume (CMV) in the blood of fish fed on dextrin and cornstarch diets compared to other experimental diets. These results indicated that low complexity carbohydrate sources induced oxidative stress and depressed growth performance. Overall, these results indicate that dietary dextrin and starch were more efficiently utilized than glucose as an energy source by juvenile Nile tilapia. This information is of increasing interest in fish nutrition to provide healthy and economically feed formulations.

## 1. Introduction

In aquafeeds, proteins are the interesting and limiting components in diets, not only in terms of cost but also in shortage of supply, especially in fishmeal, a resource that is becoming increasingly scarce in the view of the growing demand by the aquaculture sector. Indeed, the supply is decreasing and becoming significantly low, and there is no evidence to suggest a recovery in the future. Likewise, soybean meal (SBM) is considered to be the most commonly used plant protein source in fish feed formulation, especially for freshwater omnivorous fish species [1,2,3,4]. However, during the last few years, the price of SBM, has significantly increased by more than 39% from 2015 to 2018 [5]. Therefore, both ingredients are becoming prohibitively costly for their sustainable use in aquafeeds in the foreseeable future, and the cost is forecasted to be higher in the next few years, making them a non-sustainable choice for aquaculturists or aquafeed producers.

In the face of this challenge, in last few decades, substantial efforts have been oriented to identify alternatives to these ingredients (i.e., fishmeal and SBM) from unconventional sources of proteins and carbohydrates, particularly of vegetal origin, with variable success [2,6,7,8,9,10]. These attempts may not be enough if the diet profile is not improved so as to boost and maintain the good health, welfare, and immune capacity of rearing fish species, abreast of growth performance. In fact, it is well acknowledged that advancements in immuno-nutrition studies largely confirm that nutritional aspects, health conditions, and immune status of cultured fish species are closely linked. Therefore, the development of more efficient feed to enhance growth and feed efficiency and to maintain the health status of farmed fish is the most requisite for the sake of sustainability. Investigations and information depicting the effects of the interactions among diet composition and fish health and welfare allow fish nutritionists and food producers to recast and tailor their feed formulation to improve the nutritional value of the diet and hence to offer an adequate and healthy diet for fish [9,11,12,13,14,15].

Carbohydrates are the lowest-cost form of supplied dietary energy and more available than proteins. Generally, omnivorous warm-water fishes can utilize efficiently higher levels of dietary carbohydrates compared to carnivorous cold-water fishes due to higher amylase activity in the digestive tract [16] and to a higher number and higher affinity of insulin receptors [17,18]. Factors that affect the carbohydrate utilization efficiency in dietary regimes of cultured fish species are carbohydrate origin, dietary carbohydrate content in the diets, physical state, and molecular complexity. Related to the latter factor, it appears that in order to utilize simple versus complex carbohydrates, most fish species use polysaccharides and oligosaccharides better than mono and disaccharides (see review in Hemre et al. [19]). However, other species use more efficiently simple carbohydrates than complex ones such as the gilthead sea bream (*Sparus aurata*), the rainbow trout (*Oncorhynchus mykiss*), the grass carp (*Ctenopharyngodon idella*), etc. [19,20].

Some reports have found that excess inclusions or inadequate types of carbohydrates in fish diets cause metabolic stress and consequently reduce growth performance [1,3,19] and may lead to suppress immune functions and hence affect health status [21,22]. Generally, maintained hyperglycaemia after a meal reduces fish growth [23] because their tolerance to glucose is low. In fact, because glucose is more rapidly absorbed than complex carbohydrates, the excess glucose is expelled from the blood circulation instead of its metabolization in the mitochondrial hepatic cells. Besides affecting growth performance, product quality (body composition) is also affected by dietary carbohydrate level and sources through the modulation of the metabolic hepatic enzymes. Lin and Shiau [24] and Azaza et al. [1] demonstrated that carbohydrate level affects the hepatic enzymes in Nile tilapia by stimulating lipogenic enzymes. However, the carbohydrate quality effects related to these aspects are less documented and controversial.

The effects of carbohydrate complexity in some physiological aspects such as hematological parameters, digestibility, and antioxidant enzymes activities can be an useful indicators for the selection of adequate carbohydrate sources, thereby ensuring a healthy nutrition for the farmed fish species. Indeed, for example, hematological indices have been acknowledged to be valuable indicators in assessing the health state of the cultured fish in response to the dietary regime [8,25,26]. Strikingly, as indicated by Li et al. [11], “studies concerning the health implications of dietary carbohydrate in fish are still limited.” For the Nile tilapia, which is the world’s second (after carp) most popular species of farmed fish, practically no studies have been directed to the assessment of the effects of dietary carbohydrate complexity on hematological parameters and antioxidant enzymes activities. Therefore, through this study, we attempt to investigate the effects of different carbohydrate complexity, on growth performance, nutrient digestibility, feed conversion efficiency, hematological indices, and hepatic oxidative stress.

## 2. Materials and Methods

The research and procedures on Nile tilapia rearing were carried out in accordance with the local ethics committee guidelines (IACUC) at the Pasteur Institute of Tunisia, Tunisia, Tunis (IRB00005445, FWA00010074).

### 2.1. Fish and Culture System

The growth trial was carried out at the Fish-Culture Research Station of the National Institute of Marine Sciences and Technologies (INSTM). Seven-hundred and twenty six-week-old, uniform-sized fish (2.12 ± 0.02 g; mean ± SE, *n* = 720) were sampled. Only normal and visually healthy fish were selected. Efforts were made to reduce as much as possible the coefficient of variation (CV < 10%) within groups to select homogenous populations. In different dietary treatments, the mean initial body mass M did not differ significantly (one-way analysis of variance, ANOVA, F = 0.44, *p* = 0.112, df = 719.

The sorted fish were randomly dispatched to obtain 12 batches of 60 fish each and equally distributed into 12 120-L cylindrical fiberglass tanks (60 fish per batch) and kept indoors. Triplicate tanks per dietary treatment were used. Each tank was part of an open recirculation system as previously described by Azaza et al. [1,2,27]. The water was constantly equally replaced by continuous flow at a rate of 4–6 L min^−1^ tank^−1^ to provide oxygen and remove excess nitrogenous wastes, and sometimes moderate aeration was provided if necessary. The photoperiod was maintained at 12-h light and 12-h dark, with light phase start from 07:00 to 19:00.

Prior to the start of the feeding trial, fish were acclimated to the rearing system for seven days. During this period, they fed with a mixture of the four experimental diets and dead or apparently stressed fish were removed and replaced by individuals of similar sizes.

On the eve of the growth trial, the acclimated fish were starved for 24 h and individually weighed. At the beginning of the experiment, each experimental diet was allocated randomly to three tanks, thus reducing tank effect. The fish were hand-fed to apparent satiation four meals daily, five days per week (08:00, 11:00, 14:00, and 17:00) and two meals daily on weekends (09:00 and 12:00). Apparent satiation was attained when the first feed refusal was observed.

The daily feed intake (FI) per tank was recorded daily by weighing the feed at the start and at the end of each day. To avoid lost feed, feed was distributed slowly to ensure that fish ate all the offered diet. There were no wasted pellets for any of the diets during the study. The tanks were checked daily for mortalities; dead fish (if any) were removed and weighed, enabling accurate calculation of the FCR. In addition, the tanks were siphoned daily, before the first feeding, to remove sedimented fecal material and were fortnightly cleaned thoroughly by scrubbing. On cleaning days, the fish were fed only twice in the afternoon, at least 3 h after the last tank was cleaned, to allow all fish to recover from stress-related manipulation and hence to minimize the effects of this stress on FI. At the end feeding trial (day 45) and after an overnight fast, all fish in each tank were individually weighed after they were pre-anesthetized using tricaine methane sulfonate (MS-222) (100 mg L^−1^).

The water quality was daily monitored: water temperature, dissolved oxygen, and pH levels in each tank (at 20 cm below the water surface) were measured in situ with a digital thermo-oximeter surveillance system (WTW, MIQ-C184, www.memecosales.com (Lincolnwood, IL, USA); accuracy of 0.1 °C and 0.1 mg O_2_L^−1^). Total ammonium and nitrite were measured once weekly using standard methods [28]. Water temperature was maintained nearly 28 °C throughout the experiment, which corresponds to the thermal optimum one for growth of the Nile tilapia [29]. No critical values were recorded for dissolved oxygen (>4.47 mg L^−1^). Nitrite (NO_2_-N: <0.009 mg L^−1^), and total ammonia-N (TAN, NH_3_-N^+^NH_4_^+^-N: < 0.1 mg l^−1^) values remained below the limits recommended for the rearing of Nile tilapia [30,31].

### 2.2. Formulation of Feeds and Preparation

Prior to feed formulation, all feed ingredients were analyzed for their proximate composition. Based on these data and nutritional requirements of Nile tilapia [32], four isonitrogenous and isolipidic diets, with approximately 25% crude protein and 8% crude lipid, were formulated. Fish meal and SBM were used as protein sources. A 3:1 ratio mixture of soybean:cod liver oil as lipid sources was used to achieve the optimum lipid quality required for juvenile Nile tilapia [33]. Glucose, maltose, dextrin, and corn starch were incorporated at 20% as carbohydrate sources in the diets GLU-diet, MAL-diet, DEX-diet, and CSTA-diet, respectively. Before use, SBM was autoclaved at 110 °C for 30 min according to Azaza et al. [34]. Ingredients and proximate composition of the experimental diets are presented in Table 1.

The preparation of the experimental diets was based on the process described by Azaza et al. [1,2,6]. All the ingredients were finely ground (Ultra Centrifugal Mill ZM 200 Retsch GmbH, Haan, Germany) for passing through a 0.25-mm sieve. All the ingredients for each diet were fully mixed with a vitamin-mineral premix (dissolved in a small quantity of water) and oils in a food mixer (model: CAM A30, Retsch GmbH, Haan, Germany). After that, they were supplemented with 0.5% carboxymethylcellulose as a binder, 0.5% mono calcium phosphate (Ca(H_2_PO_4_)_2_) and 0.5% DL-methionine [35]. Hot water (80 °C, to accomplish agglutination) in a proportion of 40 g water/100 g diet was progressively added to the dry mix. The obtained dough was pelleted through 2.5 mm holes in a mincer (model: amb TC22SL, Oni2, Bruxelles, Belgium). The spaghetti-like strands were air-dried. The dry pellets were conserved in a freezer at −20 °C, after which they were packaged in sealed plastic bags until use. Before feeding, the dried diets were broken up and sorted to provide appropriate feed particle sizes according to the target fish size throughout the experiment, according to the model (the SGR-to-feed particle size relationship) developed by Azaza et al. [27].

### 2.3. Sampling and Chemical Analyses

At the end of the feeding trial, the feeding was discontinued for 24 h, and six fish from each tank (i.e., 18 fish per dietary treatment) were randomly sampled and anesthetized (MS-222; 200 mg L^−1^) for blood collection. Blood samples were collected from the caudal vein using heparinized syringe (5 mL), pooled, and stored in heparinized tubes at 4 °C until analyzed to determine blood indices. The same sampled fish were dissected to get liver, viscera, and mesenteric fat for the calculation of the hepatosomatic index (HSI), the viscerosomatic index (VSI), and the intraperitoneal fat ratios (IPFRs), respectively. The collected livers were washed in cold saline (0.9% NaCl), frozen immediately in liquid nitrogen, and conserved at −70 °C for hepatic antioxidant enzymes assays.

For blood analyses, hematological parameters including the red blood cells (RBCs), white blood cells (WBCs), hemoglobin (Hb, g dL^−1^), and hematocrit (Hct %) values were immediately evaluated and calculated according to Khan and Abidi [36]. Hematocrit was measured according to the microhematocrit method and hemoglobin concentration was determined using the cyanmethemoglobin technique. RBCs and WBCs were calculated using a Neubauer chamber.

The calculated blood cell indices; mean corpuscular volume (MCV), mean corpuscular hemoglobin (MCH), and mean corpuscular hemoglobin concentration (MCHC) were calculated as described by Xuezhen et al. [37].

MCV (fl, femtolitres) = (Hct/RBCs)(1)

MCH (Pg, picograms) = (Hb/RBCs)(2)

MCHC (g/dL, grams per deciliter) = (Hb/Hct)(3)

### 2.4. Antioxidant Enzyme Activity Determinations

Determination of the hepatic antioxidant enzymes was performed in liver homogenates prepared according to Saïdi et al. [7]. Livers were cut into small parts and homogenized in ice-cold phosphate buffer (50 mM Tris buffer with 150 mM KCl, 5 mM MgSO_4_, 1 mM DTT and 1 mM ethylenediaminetetraacetic acid, adjusted by HCl to pH 7.4) in a ratio of 1 g sample:5 mL buffer. The homogenate was centrifuged at 30,000× *g* at 4 °C for 25 min (Beckman GS-15R, Fullerton, CA, USA); the obtained supernatant was divided into several aliquots and stored at −80 °C, for the biochemical determinations. The assayed antioxidant enzymes were superoxide dismutase (SOD), catalase (CAT), glutathione-S-transferases (GSTs), and glutathione peroxidase (GPx). The soluble protein content of the liver homogenates was determined as described by Bradford [38] against bovine serum albumin (BSA) as standard. The absorbance of samples was measured at 595 nm.

SOD activity was monitored spectrophotometrically at 550 nm, through the photoreduction of nitroblue tetrazolium (NBT) [39]. One unit of SOD activity is defined as the amount of enzyme able to inhibit 50% of the control rate of cytochrome c reduction.

CAT activity was measured at 25 °C according to Aebi [40] every 30 s for 2 min. The reaction mixture (1 mL) contained 100 mM phosphate buffer (KH_2_PO_4_; pH = 7), 500 mM H_2_O_2_ and the erythrocyte homogenate. The enzyme activity was expressed as IU mg^−1^ protein using a molar extinction coefficient of 40 M^−1^ cm^−1^ [41].

GSTs activity was determined by following the formation of glutathione-1-chloro-2, 4-dinitrobenzene (CDNB) adducts at 340 nm. The conjugated CDNB (mmoles) were calculated according to Habig et al. [42].

GPx activity was calculated by following the rate of NADPH oxidation at 340 nm by a coupled reaction with glutathione reductase [43]. Enzymatic activity is expressed as nanomoles of NADPH extinction per minute per milligram of protein. All enzyme assays were performed in sextuplicate.

### 2.5. Digestibility Trial

The remaining fish (42–50 per tank) were used for the digestibility trial. In vivo, apparent digestibility coefficients (ADC_S_) of the experimental diets were evaluated in a separate trial using an indirect method. Fish were fed with the same experimental diets as in the first experiment, except that 0.5% chromic oxide (Cr_2_O_3_) was added to each diet as a non-absorbable indicator (i.e., external indigestible marker). The same feeding schedule, as in the feeding trial, was adopted, and fish were fed for 10 days before any fecal samples were collected. This enable fish to adapt to the chromic-oxide diets. At day 10, ten fish from each tank were sampled, killed using over-dose of tricaine methane sulfonate (200 mg L^−1^) and block weighted to ensure whether growth performance followed the same trends as in the growth trial. The collection and preparation of fish feces for chemical analysis were determined as previously described by Azaza et al. [1,2] using the dissection method [44]. In fact, feces were taken from posterior intestine, 8 h after feeding, centrifuged (4000 rpm, 15 min), lyophilized, finely ground with an ultrafine pulverizer, and conserved in a freezer at −20 °C.

ADCs were determined for dry matter, fat, protein, and carbohydrate. For digestibility assessment, chromic oxide concentration of the feed and fecal samples, in triplicates, was estimated by the method of Furukawa and Tsukahara [45] based on the acid digestion technique.

The ADCs were calculated using the following formula:

ADC Dry matter (%) = 100 [(1 − (D_y_/F_y_)] (4)

ADC_i_ = 100 [1 − (F_i_/D_i_) (D_y_/F_y_)] (5)

where F_i_ and D_i_ were the percentages of nutrient in the feces and diet, respectively, and F_y_ and D_y_ were the percentages of chromic oxide in the feces and diet, respectively. The chemical analyses of the experimental diets and sampled feces were performed, in triplicate, using the following procedures as described by AOCA [46]: dry matter (dried at 105 °C until a constant weight was achieved), crude protein by the Kjeldahl method after an acid digestion (6.25 nitrogen to protein conversion factor), crude lipid (ether extract according to the soxhlet method) and ash (muffle furnace, oven incineration at 550 °C for 6 h). Carbohydrate (as NFE) was calculated as follows: NFE = 100 − (% protein +% lipids + % ash +% fiber. Gross energy was estimated based on the following conversion factors: carbohydrate (as NFE): 17.2 MJ kg^−1^, protein: 23.6 MJ kg^−1^ and fat: 39.5 MJ kg^−1^ [47]. Digestible energy content was estimated according to Morais et al. [48], using the following conversion values: 23.6 MJ kg^−1^ for protein, 39.5 MJ kg^−1^ for lipid, and 17.2 MJ kg^−1^ for carbohydrate and average nutrients digestibility values of 90%, 85%, and 50%, respectively, for protein, fat, and carbohydrate.

### 2.6. Calculations and Statistical Analyses

Fish growth was expressed as the SGR (% M day^−1^), which was calculated as SGR = 100 (lnM_f_ − ln M_i_)/(t_x_ − t_1_); where ln is log_e_ and M_x_ and M_i_ are the mean body masses of fish at times t_x_ and t_1_, respectively. The FCR (g/g) was calculated as FCR = FI/(Bf − Bi + Md); where Bi and Bf are the total body masses (g) of fish at the start and end of the experiment, Md_x-1_ is the biomass of fish dying throughout the experiment, and FI (g) is the quantity of distributed feed during the rearing period. PER was calculated as follows: PER = (wet mass gain, g)/(protein intake, g).

HIS, VSI, and IPFRs were calculated as follows:

HIS = 100 (liver wet mass)/(body wet mass);(6)

VSI = 100 (viscera wet mass)/(body wet mass);(7)

IPFRs = 100 (IPF wet mass/whole body mass).(8)

All data were analyzed for normal distribution by Shipiro–Wilk tests [49] and for homogeneity of variances by Barlett’s test [50]. Arcsine transformations of percentage data and log transformations for other data were performed to achieve homogeneity of variance. Data that were normal and homogeneous was analyzed by one-way ANOVA using the software of Statistica^®^ version 5.1 (Statsoft Inc, Tulsa, USA). When significant differences were detected, Duncan’s multiple-range tests (DMRT) was used for post hoc analysis. All differences were considered to be significant at a probability level of 0.05. Values are expressed as mean ± error standard of means of three or six replicates (ESM; *n* = 3 or 6).

## 3. Results

### 3.1. Survival, Feed Intake, Growth Performance, and Feed Utilization

Feed formulation and proximate composition of the experimental diets are presented in Table 1. Data on SGR, FCR, and PER of the juvenile Nile tilapia after 45 days of feeding trial are presented in Table 2. At the end of the growth trial, survival rates of fish were greater than 90% and not affected by dietary treatment. Feed acceptance was satisfactory and seems identical for all groups, with no observed rejection. Fish manifested an active feeding behavior, and FI increased in fish from all the tanks during the course of the experiment. There was no significant difference in FI among fish fed with the MAL-diet, DEX-diet, and CST-diet (*p* > 0.05, Table 2). However, fish fed with GLU-diet depicted higher (*p* < 0.05) FI than that of fish fed with the other experimental diets.

The final body mass and SGR of groups fed on MAL-diet, DEX-diet, and CST-diet did not differ significantly, whereas a significant (*p* < 0.05) decrease in growth was observed in groups fed on GLU-diet. The FCR were ranged from 1.49 to 1.94. Only groups fed on the GLU-diet had significantly (*p* < 0.05) lower feed conversion efficiency (Table 2). The PER in fish fed with the GLU-diet was significantly lower than those fed with the other diets (*p* < 0.05), which were equal among themselves.

### 3.2. Nutrients Digestibility

The nutrients digestibility of the experimental diets is presented in Table 3. The dry matter digestibility was higher (*p* < 0.05) in groups fed on dextrin and cornstarch diets. The same trend was also observed for carbohydrate digestibility. The ADCs of carbohydrate ranged between 79.73 and 74.97% and were significantly lower in fish fed on cornstarch diet. However, the digestibility of crude protein and energy did not differ significantly (*p* > 0.05) among the groups.

### 3.3. Slaughter Indices

The effects of the dietary regimes on slaughter indices are presented in Table 4. Fish fed with dextrin and cornstarch diets had significantly higher VSI values than those fed with the other diets, and the lowest VSI values was observed in groups fed with maltose and glucose diets. There were no significant differences in the HSI values among the groups fed with, glucose, maltose and dextrin diets, while fish fed with cornstarch diet had the highest HSI value. The same trends were observed in IPFR.

### 3.4. Hematology and Hepatic Antioxidant Enzymes

Some hematological indices such as Hct, Hb, red blood cells count (RBC), white blood cells count (WBC), mean cellular hemoglobin (MCH), mean cellular hemoglobin concentration (MCHC), and MCV in the blood of juvenile Nile tilapia at the end of feeding trial are indicated in Table 5. There was a significant increase in Ht, Hb, and CMV in blood fish fed with the dextrin and cornstarch diets compared to fish fed the other experimental diets. However, there was an insignificant effect of dietary treatment on RBC, MCH, and MCHC. The WBCs level significantly increased in fish fed with corn starch-containing diet compared to the other groups (*p* < 0.05).

**Figure 1:**
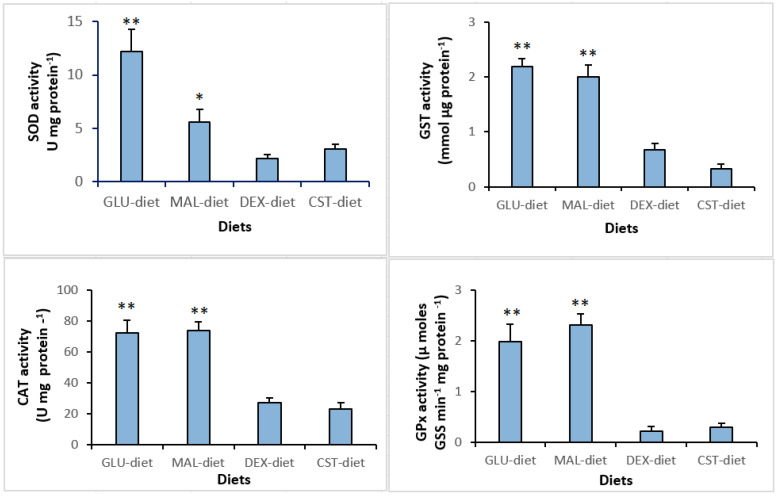
Specific activities of superoxide dismutase (SOD), glutathione S-transferase (GST), catalase (CAT), and glutathione peroxidase (GPx) in the livers of juvenile Nile tilapia O. niloticus fed on experimental diets. In each graph, bars and whiskers represent the means + SEM. Values represent the mean of six measurements (*n* = 6). Significantly different values are indicated by asterisks (* *p* < 0.05; ** *p* < 0.01).

The activities of antioxidant enzymes in the liver are presented in Figure 1. Results indicate that superoxide dismutase (SOD), glutathione S-transferase (GST), catalase (CAT), and glutathione peroxidase (GPx) activities were significantly affected by dietary treatments. CAT, GST, and GPx activities were significantly (*p* < 0.01) higher in the livers of groups fed on glucose and maltose containing diets when compared to other groups. Regarding SOD activity, fish fed on glucose and maltose diets have significantly (*p* < 0.01 and *p* < 0.05, respectively) higher values compared to other dietary regimes.

## 4. Discussion

Proteins are the costliest components of formulated aquafeed, thus, the use of alternative ingredients that diminish feed costs is the main priority to reduce effectively the fish cost production [1,2]. There is a growing interest in using lipid or carbohydrate as energy source, then sparing proteins [51,52] and reducing the diets’ cost. In fact, carbohydrate rich ingredients were largely considered as the cheapest form of dietary energy and efficiently harnessed by herbivorous or/and omnivorous fish species. These species, compared to carnivorous fish, present high amylase activity and insulin receptors. However, their aptness to utilize dietary carbohydrates varies among the species and even into the same species. Many factors may be involved in the diversity of the ability to utilize carbohydrate such as body size, dietary composition, gastrointestinal anatomy, feeding schedule, rearing water temperatures, hormonal response etc. In addition, carbohydrate molecular complexity is largely considered as a predominant factor, but controversies still remain. Thus, it is difficult to infer behavior for any species or its life stage, without experimental validation. Besides, inappropriate dietary carbohydrate content (level and/or quality), may result in physio-pathological disturbance in fish [19] such as glycogen deposition, hepatocyte hypertrophy, metabolic disturbance, metabolic stress etc. Consequently, these aspects might adversely affect growth performance and feed efficiency, with suppression of the immune functions and increase of susceptibility to infectious diseases [21]. This is the first study attempting to investigate the effects of carbohydrate complexity on the growth performance, hematological parameters and oxidative stress of the Nile tilapia.

As presented above, the results of the present study demonstrate that growth performance and feed efficiency seem to be positively correlated to carbohydrate complexity. The lower growth and feed utilization efficiency of Nile tilapia fed a glucose-containing diet than those fed the diets containing dextrin and corn starch in the present study are similar to those reported for other fish species such as channel catfish [53], starry flounder [54], yellowfin sea bream [55], and cobia [56]. However, Hung et al. [57] reported that the growth performance of white sturgeon fed monosaccharides and disaccharides-based diets was significantly higher than that fed on starch-based diet. Similarly, Enes et al. [58] demonstrate that gilthead sea bream utilizes more efficiently low complexity carbohydrate sources than dextrin and starch-based diets. An intermediate situation that growth performance was unaffected by carbohydrate complexity (dietary glucose, dextrin, and starch) have been reported in red sea bream [59] and grouper [60].

The reduction of growth performance and feed conversion efficiency of the groups fed on glucose and maltose diets could be related to the faster assimilation of the glucose in the digestive tracts [58]. As a result, the absorbed excess of glucose may be disburdened from blood (through excretion processes) before its metabolic use. In fact, fish fed the glucose and maltose diets had lower FCR and PER values compared to those fed with other diets. This reduction of the PER clearly indicates that more quantity of protein was catabolized and used for energy purposes, rather than for somatic growth.

Regarding nutrient digestibility, results showed that the low complexity carbohydrate-based diets were associated with a reduction in ADC of dry matter and protein with concomitant increase in daily FI (Table 3). The daily FI of fish fed with GLU-diet (the lowest carbohydrate complexity based-diet) was significantly higher than those fed with the other experimental diets. This increase in FI might be due to limited energy availability from inefficient digestibility. This finding is reported in some species of aquaculture interest such as the gilthead sea bream [61,62], sea bass [63], the hybrid *Oreochromis niloticus* x *O. aureus* [64], and Nile tilapia [1,6] in which the FI can be adjusted in fish fed to satiation to meet their energy requirements. The opposite was observed in cobia [54] in which less FI of the fish fed the glucose diet, compared to groups fed with more complex carbohydrate-based diet.

The assessed slaughter indices (HSI, VSI, and IPFR) were significantly influenced by the dietary carbohydrate sources. Indeed, fish fed with glucose and maltose diets presented significantly lower HSI and VSI indices than groups submitted to other dietary treatments. These results are in disagreement with those reported for other species such as yellowfin seabream [55], hybrid striped bass [65] and cobia [56]. However, some previous studies have shown that carbohydrate not used for energy purposes can be accumulated in the liver as fat and glycogen [1,66]. This process can enhance HIS and VSI indices. Our results are in agreement with previous ones observed in the hybrid tilapia *O. niloticus* × *O. aureus* [64] and other species [19,52,53]. This can be assigned to increased glycogen deposition [19,67]. Also, increased liver fat deposition has been observed in other fish such as cod [68] and cobia [56]. These authors have shown that lipid and glycogen bulks in the liver were higher in juvenile cobia fed with diets containing dextrin and corn starch than in those fed with diets containing glucose, sucrose and maltose. In fact, Mohanta et al. [69] and Fernandez et al. [52] proposed that the increase in the carcass lipid with the increase in dietary carbohydrate could be explained by de novo synthesis of lipid from carbohydrate. These assumptions have been shown in juvenile Nile tilapia [1], in which a significant increase activity of the pentose phosphate pathway regulatory enzymes is observed. That means an increase in the glycolytic capacity and NADPH generation, necessary for transformation of glucose into fat.

Generally, the assessment of hematological parameters is a valuable tool for diagnosing diseases, fish health status and qualifying nutrients. Osuigwe et al. [70] reported that in fish, the hematological parameters are likely to be affected by a multitude of factors such as fish species, life stage, environmental rearing conditions and dietary regime. In the present study, fish fed with the glucose and maltose diets depicted in general lower Ht, Hb, and WBC values than those fed with the other diets. The white blood cell value is considered to be an excellent indicator of fish health, associated with the presence of infection, and with the type of response to it or to some other physiological or pathological factor [71]. These negative effects observed in the groups fed on glucose and maltose-diets were more likely due to the nutritional stress of the juvenile Nile tilapia fed with the low complexity carbohydrate-based diet. It has been reported that these such behavior is related to disturbances observed in fish health [72]. On the other hand, an improvement of the hematological indices was recorded in fish fed on dextrin or corn starch diet, which is, presumably, the outcome of a good health condition.

The negative effects of reducing Hb in fish fed with a staple diet based on glucose could be explained by the low Hb synthesis due to the weak iron assimilation [21,23]. In fact, several fish species, present a reduction of iron absorption in consequence of a hyperglycemia associated with increased dietary suggar [73,74]. Besides, pronounced fall of Hb content may reflect the capacity of metabolic regulation in response to high oxygen availability [75]. WBC have a significant role in the setting of the innate immunity, and their count gives a reliable indication of fish health status [8,10,12,76]. In the present study, juvenile Nile tilapia fed on glucose containing diet depicted relatively low WBC number, presumably indicating an immuno-suppression in these fish.

In physiological homeostasis conditions, animal cells generate reactive oxygen species (ROS) while at the same time maintaining several antioxidant defenses through an opposite set of mechanisms toward equilibrium. The disruption of this balance between the two prosses, an oxidative stress can be produced [77]. Although it seems obvious that the antioxidant defense mechanisms depend directly on nutritional staple [78], there is little information on how the level and nature of dietary nutrients affect the oxidative status in fish [79]. The present study shows that the liver antioxidant assayed enzymes CAT, SOD, GST, and GPx were responsive to the presence of low complexity carbohydrate (i.e., glucose and maltose) in the diets. This metabolic response suggests that a higher oxidation rates occurs in tilapia fed on glucose and maltose-based diets. These enzymes are commonly used in toxicological assessment as oxidative stress indicators [3,79]. Therefore, the increased activities observed in such enzymes can be interpreted as oxidative stress. On the opposite side, the lowest antioxidant enzyme activities were observed in groups fed on dextrin and cornstarch diets, indicating that these dietary regimes are unlikely to lead oxidative stress of juvenile Nile tilapia. Although we have not estimated oxidative damage at this stage such as lipid peroxidation (TBARS) and protein carbonyl levels as indicators, we have been able to establish a correlation tendencies between carbohydrate complexity and analyzed enzymes activities (SOD, CAT, GST, and GPx), an indicator of susceptibility to oxidative stress.

Several processes related to carbohydrate metabolization appear to be implicated in the induction of this oxidative stress via various cytotoxic roles for glucose such as self-oxidation (enolization), glycosylation, and especially through the polyol pathway mechanisms [80]. For instance, products derived from the autoxidation of glucose, called ketoaldehydes analogous, can adhere to amino acids groups of proteins and lead to monosaccharide attachment and protein oxidative impairment which at the end could adversely affect the oxidative status [76].

Glycosylation is a pathological aspect, occurring under conditions of hyperglycemia, giving rise to glycated proteins. The first step in glycation corresponds to the reaction between low carbohydrate complexity (e.g., glucose, fructose) and the free amine function of a protein to form a Schiff base. This reaction is followed by rearrangements which will give rise to the formation of an intermediary products which undergo numerous oxidative reactions, thereby, generating reactive oxygen species. Moreau et al. [81] demonstrated that Nile tilapia, like other omnivorous species, is characterized by its glucose intolerance, marked by the postprandial persistent hyperglycemia. Since the glucose concentration is maintained as higher after the meal, it can be assumed that the glycation of proteins will be more important.

Regarding the polyol pathway mechanisms, the physiological state, marked by the hyperglycemia can cause part of the glucose to be diverted to the polyol pathway [82]. Under normal physiological conditions, the polyol pathway is inactive. However, when glycemia increases, some of the glucose is reduced to sorbitol by the action of aldose reductase, using NADPH as co-factor [76]. Sorbitol will build up in cells due to its inability to cross membranes and cause multiple damage such as osmotic damage. Part of the sorbitol can be oxidized to fructose causing advanced glycation products, acknowledged as a potential source of oxidative stress by generating oxygen free radicals [83]. In addition, the use of NADPH, as a co-factor, will decrease its availability for glutathione reductase activity, important for the formation of reduced glutathione [79]. The activation of the polyol pathway will therefore lead to an increase in oxidative stress within the cell with a decrease in antioxidant defenses.

## 5. Conclusions

In conclusion, data from the present study could have practical implications for the optimization and tailoring of practical diet formulations. All in all, the results indicate that juvenile Nile tilapia can efficiently utilize dextrin and corn starch as carbohydrate energy sources. However, dietary glucose and maltose reduce growth performance, feed utilization efficiency, and adversely affect the health status of the juvenile Nile tilapia. These findings allow aquaculture nutritionists to recast and adjust their feed formulation accordingly.

## Figures and Tables

**Table 1:** Ingredients and proximate composition of the experimental diets (% dry matter).

Components	Experimental Diets			
	GLU-Diet	MAL-Diet	DEX-Diet	CST-Diet
**Ingredients (% g kg^−1^)**				
Fish meal	170	170	170	170
Soybean meal (DSBM) ^a^	270	270	270	270
Maize meal	250	250	250	250
Glucose	200			
Maltose		200		
Dextrin			200	
Corn starch				200
Soybean oil/cod liver oil (3:1) ^b^	75	75	75	75
Vit-mineral mix ^c^	10	10	10	10
CMC (binder) ^d^	10	10	10	10
Monocalcium phosphate ^e^	5	5	5	5
L-methionine	5	5	5	5
Chromic oxide ^f^	5	5	5	5
**Proximate analysis (% g kg^−1^) ^g^**				
Dry matter (in original matter)	881.7	928.7	917.3	890.4
Crude protein	251.4	250.6	254.3	259.1
Crude lipid	111.9	106.0	115.1	124.3
Crude fibre	66.6	58.7	60.9	68.3
Ash	5.21	61.3	54.7	60.2
Carbohydrate ^h^	518.0	523.4	515.0	488.1
Gross Energy (MJ kg^−1^ DM) ^i^	18.91	18.76	19.05	19.04
Digestible Energy (MJ kg^−1^ DM) ^j^	13.55	13.38	13.69	13.87

^a^ DSBM was autoclaved at 110 °C for 30 min according to Azaza et al. [34]. ^b^ mixture of soybean oil (SO) and fish oil (FO); (ratio; SO:FO = 3:1 according to El–Husseiny et al. [33]. ^c^ Vitamin premix and mineral premix were described in Azaza et al. a and b [1,2]. ^d^ CMC = carboxymethylcellulose. ^e^ Ca(H_2_PO_4_)_2_^f^ Cr_2_O_3_; inert marker, used only for digestibility trial. ^g^ Dry base ^h^ N-free extract calculated by difference = 100 − (crude protein + crude lipid + crude fiber + ash) ^i^ Calculated using the factors: carbohydrates, 17.2 kJ g^−1^; protein, 23.6 kJ g^−1^ and, lipids, 39.5 kJ g^−1^ [47]. ^j^ Estimated DE = (crude protein × 23.6 × 0.9) + (crude lipid × 39.5 × 0.85) + carbohydrate × 17.2 × 0.5) [48]. GLU-diet: glucose, MAL-diet: maltose, DEX-diet: dextrin, and CST-diet: corn starch.

**Table 2:** Growth performance, feed utilization efficiency and biological parameters of juvenile Nile tilapia fed experimental diets. Each value is a mean ± SEM derived from three replicates, (*n* = 3 tanks per diet) ^a^.

Variables	Experimental Diets			
	GLU-Diet	MAL-Diet	DEX-Diet	CST-Diet
IBM (g)	2.28 ± 0.1	2.16 ± 0.09	2.11 ± 0.07	2.22 ± 0.08
FBM (g)	24.48 ± 1.46 ^a^	31.12 ± 1.33 ^b^	30.48 ± 1.69 ^b^	28.93 ± 1.82 ^b^
SR (%)	92.78 ± 0.67	91.64 ± 3.47	90.00 ± 1.16	93.33 ± 2.00
SGR (% day^−1^)	5.27 ± 0.08 ^a^	5.93 ± 0.18 ^b^	5.96 ± 0.09 ^b^	5.71± 0.16 ^b^
FI (g day^−1^)	51.44 ± 2.61 ^a^	48.68 ± 1.77 ^ab^	42.95 ± 2.49 ^b^	43.43 ± 2.89 ^b^
FCR (g g^−1^)	1.94 ± 0.19 ^a^	1.66 ± 0.20 ^b^	1.49 ± 0.12 ^c^	1.55 ± 0.15 ^b,c^
PER (g g^−1^)	2.01 ± 0.13 ^a^	2.27 ± 0.18 ^ab^	2.67 ± 0.15 ^c^	2.54 ± 0.14 ^c^

^a^ Values in the same row followed by different superscripts letters (a, b and c) are significantly different (DMRT, *p* < 0.05). IBM, initial body mass; FBM, final body mass; SR, survival rates; SGR, specific growth rate; FCR, feed conversion ratio; FI, feed intake; PER, protein efficiency ratio.

**Table 3:** Apparent nutrient digestibility coefficients (ADCs) of experimental diet components.

ADC (%)	Experimental Diets			
	GLU-Diet	MAL-Diet	DEX-Diet	CST-Diet
Dry mater	73.55 ± 3.17 ^a^	71.08 ± 2.25 ^a^	78.71 ± 2.04 ^b^	77.67 ± 2.21 ^b^
Protein	82.12 ± 3.19 ^a^	89.33 ± 2.78 ^b^	85.67 ± 2.14 ^b^	88.03 ± 2.78 ^b^
Fat	90.43 ± 2.79	87.16 ± 2.65	90.72 ± 2.98	86.04 ± 3.16
Carbohydrate	79.73 ± 2.98 ^a^	75.44 ± 2.42 ^a^	77.86 ± 2.89 ^a^	74.97 ± 3.11 ^b^
Energy	81.83 ± 3.04	79.44 ± 2.67	82.67 ±1.97	80.38 ± 2.27

Data are expressed as mean ± SEM (*n* = 3). Values on the same line and with different superscripts letters (a and b) are significantly different (*p* < 0.05).

**Table 4:** Slaughter indices (HIS, VSI, and IPFR; % of body mass (BM)) of juvenile Nile tilapia fed the experimental diets.

Variables	Experimental Diets			
	GLU-Diet	MAL-Diet	DEX-Diet	CST-Diet
HSI (% BM) ^1^	2.75 ± 0.16 ^a^	2.62 ± 0.14 ^a^	2.88 ± 0.15 ^a^	3.16 ± 0.12 ^b^
VSI (% BM) ^2^	6.94 ± 0.52 ^a^	6.81 ± 0.94 ^a^	7.92 ± 0.48 ^b^	7.83 ± 0.71 ^b^
IPFR (% BM) ^3^	1.63 ± 0.65 ^a^	1.45 ± 0.14 ^a^	1.92 ± 0.48 ^a^	2.43 ± 0.62 ^b^

Values are means ± standard error (*n* = 30 fish per dietary treatment; 10 from each tank). Values marked with different letters (a and b) within the same row are significantly different (*p* < 0.05). ^1^ Viscerosomatic index; ^2^ Hepatosomatic index; ^3^ Intraperitoneal fat ratio.

**Table 5:** Hematological characteristics of juvenile Nile tilapia fed the different experimental diets.

Parameters	Experimental Diets			
	GLU-Diet	MAL-Diet	DEX-Diet	CST-Diet
Hematocrit (Hct %)	23.15 ± 0.59 ^a^	24.08 ± 1.54 ^a^	25.71 ± 1.87 ^b^	28.67 ± 1.36 ^b^
Hemoglobin (Hb, g/dL)	6.02 ± 0.20 ^a^	5.93 ± 0.11 ^a^	9.67 ± 0.10 ^b^	10.93 ± 0.13 ^b^
RBCs ^a^ (×10^6^/mm^3^)	32.13 ± 3.9	30.56 ± 4.5	29.42 ± 4.7	37.08 ± 3.3
WBCs ^b^ (×10^3^/mm^3^)	41.63 ± 2.78 ^a^	45.63 ± 4.17 ^a^	43.86 ± 1.78 ^a^	52.91 ± 3.22 ^b^
MCH (pg) ^c^	101.4 ± 3.63	107.19 ± 1.80	104.38 ± 2.77	110.91 ± 2.85
MCHC (g/dL) ^d^	32.25 ± 4.02	26.82 ± 3.90	31.90 ± 3.34	35.63 ± 2.96
MCV (fl) ^e^	729.51 ± 14.51 ^a^	797.95 ± 8.86 ^a^	918.88 ± 9.37 ^b^	907.27 ± 12.54 ^b^

^a^ RBCs: red blood cells count. ^b^ WBCs: white blood cells count. ^c^ MCH: mean cellular hemoglobin = (Hb/RBCs). ^d^ MCHC: mean cellular hemoglobin concentration = (100 Hb/Hct). ^e^ MCV: mean corpuscular volume = (Hct/RBCs), expressed in femtolitre (fl).
